# Tailored treatment options for patients with psoriatic arthritis and psoriasis: review of established and new biologic and small molecule therapies

**DOI:** 10.1007/s00296-016-3436-0

**Published:** 2016-02-18

**Authors:** Sarah Elyoussfi, Benjamin J. Thomas, Coziana Ciurtin

**Affiliations:** University College London Medical School, Gower Street, London, Greater London WC1E 6BT UK; Department of Rheumatology, University College London Hospital, 3rd Floor Central, 250 Euston Road, London, NW1 2PG UK

**Keywords:** Psoriatic arthritis, Psoriasis, Biologic treatments, Small molecule inhibitors, Level of evidence of biologic agents efficacy

## Abstract

The diverse clinical picture of PsA suggests the need to identify suitable therapies to address the different combinations of clinical manifestations. This review aimed to classify the available biologic agents and new small molecule inhibitors (licensed and nonlicensed) based on their proven efficacy in treating different clinical manifestations associated with psoriasis and PsA. This review presents the level of evidence of efficacy of different biologic treatments and small molecule inhibitors for certain clinical features of treatment of PsA and psoriasis, which was graded in categories I–IV. The literature searches were performed on the following classes of biologic agents and small molecules: TNF inhibitors (adalimumab, etanercept, infliximab, golimumab, certolizumab), anti-IL12/IL23 (ustekinumab), anti-IL17 (secukinumab, brodalumab, ixekizumab), anti-IL6 (tocilizumab), T cell modulators (alefacept, efalizumab, abatacept, itolizumab), B cell depletion therapy (rituximab), phosphodiesterase 4 inhibitor (apremilast) and Janus kinase inhibitor (tofacitinib). A comprehensive table including 17 different biologic agents and small molecule inhibitors previously tested in psoriasis and PsA was generated, including the level of evidence of their efficacy for each of the clinical features included in our review (axial and peripheral arthritis, enthesitis, dactylitis, and nail and skin disease). We also proposed a limited set of recommendations for a sequential biologic treatment algorithm for patients with PsA who failed the first anti-TNF therapy, based on the available literature data. There is good evidence that many of the biologic treatments initially tested in psoriasis are also effective in PsA. Further research into both prognostic biomarkers and patient stratification is required to allow clinicians the possibility to make better use of the various biologic treatment options available. This review showed that there are many potentially new treatments that are not included in the current guidelines that can be used for selected categories of patients based on their disease phenotype, clinician experience and access to new biologic therapies.

## Introduction

Psoriatic arthritis (PsA) is a heterogeneous disease, which shares characteristic clinical features (sacroiliitis, spondylitis, enthesitis, psoriasis, uveitis), genetic markers and positive family history with the larger group of seronegative spondyloarthropathies. The clinical presentation can also be undistinguishable from that of rheumatoid arthritis (RA), especially in patients who have PsA with peripheral involvement. The diverse clinical picture of PsA suggests the need to identify suitable therapies to address different combinations of clinical manifestations [1]. Patients will experience a decreased quality of life as a consequence of pain, functional impairment, cosmetic implications of skin and nail lesions, and (in some cases) because of side effects to medication. The aspect of functional preservation, prevention of irreversible damage and minimisation of risk of co-morbidities are long-term goals for modern therapy in PsA [2].

Tailoring the available treatment options according to the disease phenotype is needed to ensure the use of a minimal combination of drugs for a maximal therapeutic effect. Conventional treatments for PsA have limited efficacy for nail disease, enthesitis or axial involvement, and some are unable to control moderate and severe peripheral joint and skin disease [3]. For the first time, the introduction of biologic treatments offered the possibility of controlling multiple aspects of these diseases using a single drug, minimising the need for additional therapies. At present, the overarching principle of choosing a treatment target based on a shared decision between rheumatologists and other specialists (such as dermatologists, ophthalmologists, gastroenterologists) seems more achievable. This is because many of the available biologic treatments are used for several indications across different specialties.

Here we reviewed the evidence regarding the efficacy of biologic agents for psoriasis and PsA treatment. The purpose of this was to generate a comprehensive summary of efficacy of biologic treatments for different clinical features of patients with PsA and psoriasis, such as axial disease, peripheral joint involvement, dactylitis, enthesitis, and nail and skin disease.

## Biologic agents

### TNF inhibitors

Adalimumab is a human monoclonal antibody with a high affinity for TNFα. Adalimumab is licensed for use in adults with severe psoriasis and PsA in whom conventional therapies have failed or are not tolerated.

Evidence of its efficacy in treating both psoriasis and PsA is available from numerous RCTs. Different outcome measures were improved in the treatment arms, such as Psoriasis Area and Severity Index (PASI75) [4], American College of Rheumatology (ACR) responses and PsA Response Criteria (PsARC), together with Health Assessment Questionnaires (HAQ), Health Assessment Questionnaire Disability Index (HAQ-DI), Short form-36 health survey (SF-36**),** Dermatology Life Quality Index (DLQI) score, Mental Component Summary Score (MCSS) and Functional Assessment of Chronic Illness Therapy (FACIT) fatigue scale [5–8]. Radiographic progression as measured by the modified total Sharp score at weeks 24 and 48 was lower in those treated with adalimumab irrespective of whether they were receiving methotrexate (MTX) at baseline [5, 8].

Adalimumab has also demonstrated its superiority when compared to conventional therapies, such as methotrexate and cyclosporine [9, 10]. In addition, combination of DMARDs and adalimumab also showed superiority to monotherapy [10].

Adalimumab has been compared directly and indirectly with other drugs in the TNF inhibitor group (infliximab, etanercept, adalimumab and golimumab) in patients with PsA [11–13]. All treatments have demonstrated similar outcomes and safety profiles. There is also evidence of additional benefit when switching from one anti-TNF drug to another [14, 15].

The clinicians’ choice for a biologic therapy in a particular patient may be guided by the drug ability to tackle specific manifestations of these diseases, such as axial disease, dactylitis, enthesitis and nail disease. Adalimumab is effective for the treatment of dactylitis and enthesitis [16]. One RCT [17] and three observational studies have shown effectiveness of adalimumab in nail disease as assessed by Nail Psoriasis Severity Index (NAPSI) [18–20]. A recent publication by the medical board of the National Psoriasis Foundation has recommended the use of adalimumab in patients with nail disease alone, skin and nail disease or for patients with a combination of nail, skin and joint disease [21]. Adalimumab was ranked with the “highest enthusiasm” compared to all other drugs recommended for nail psoriasis.

Adalimumab showed improvement of axial disease in patients with ankylosing spondylitis (AS), regardless of concomitant presence of psoriasis [22]. In summary, adalimumab has shown clear benefits in joint and skin disease. Studies have shown a clear reduction in disability and improved quality of life. Adalimumab may also be the drug of choice for patients with dactylitis, enthesitis and nail disease. It may also be of use in patients in whom MTX is ineffective, or other TNF inhibitors have failed, or in combination with cyclosporine [23].

Etanercept is a fusion protein consisting of the p75 receptor bound to the Fc region of human immunoglobulin (Ig) G1. It has shown efficacy at 12 weeks for PsARC, ACR20, ACR50 and ACR70, PASI75 response criteria and improvement in the quality of life, patient rating of pruritus and patient global assessment of psoriasis and physician global assessment (PGA) [24–31]. It was also shown to inhibit radiographic progression at 12 and 24 months [32, 33]. A meta-analysis assessing etanercept efficacy in comparison with other TNF inhibitors has found a lower relative risk (RR) for a PASI75 response at week 12, as well as a lower RR for PASI75 at 24 weeks than for adalimumab, golimumab and infliximab [3].

An observational study looking at patients with PsA with axial disease found that 72 % of patients had an improved Bath Ankylosing Spondylitis Disease Activity Index (BASDAI) score whilst taking etanercept [34].

Etanercept is an effective treatment for enthesitis and dactylitis, with improvements documented at week 12 and week 24 in a multiple-dose study [35]. Similar efficacy has also been demonstrated in psoriatic nail treatment [36, 37]. Etanercept is currently recommended by the medical board of the National Psoriasis Foundation for use in isolated nail disease, skin and nail disease, and nail and skin and joint disease [21].

In summary, etanercept has proven efficacy in skin and joint disease as well as nail disease, dactylitis and enthesitis, and may relieve symptoms of fatigue and depression, although, as detailed above, it appears as it is less effective than other anti-TNF drugs.

Infliximab is a chimeric monoclonal antibody against TNFα, with demonstrated efficacy for treating psoriasis [38–41]. In parallel, the drug has also been proven effective in PsA. The IMPACT RCT demonstrated significant ACR20 response at week 16 [39], and additional improvement in quality of life as assessed by HAQ score and SF-36 health survey at week 14 [42] and week 16 [39]. The efficacy in improving PASI75, PASI90, ACR20, ACR50 and ACR70 responses was sustained at week 54, regardless of baseline methotrexate use [43]. Infliximab significantly inhibited progression of radiographic damage at week 24 [44].

The EXPRESS RCT found significant improvement in nail disease for  % improvement in NAPSI score and nail matrix and bed features at weeks 10 and 24 (26.8 and 57.2 %, respectively, in the infliximab group versus −7.7 and −4.1 %, respectively, in the placebo group, both *p* < 0.001). The IMPACT RCT found significant improvement in dactylitis and enthesitis scores at week 16 [39], and these were maintained until week 54 in the IMPACT-2 RCT [43].

Infliximab has demonstrated superiority compared to conventional treatments, as assessed by both PsA and psoriatic outcome measures [45, 46].

Infliximab has also demonstrated efficacy in psoriasis for patients with an inadequate response to etanercept: in the PSUNRISE RCT, at week 10, 65.4 % of patients had achieved a PGA score of 0 or 1 (indicating clear or almost clear disease), and this was sustained until week 26: 61.3 % [47].

Certolizumab is a PEGylated Fab’ fragment of a humanised TNF inhibitor monoclonal antibody, which has been proven beneficial in treating psoriasis [48] and PsA. Certolizumab has also been associated with preserved efficacy in patients with previous exposure to TNF inhibitors [49], sustained effectiveness [50], and additional benefit in improving the quality of life [51] and productivity [52]. In addition, there was significant inhibition of radiographic progression as measured by the modified total Sharp score at week 24 [53].

Certolizumab is also effective in treating enthesitis, dactylitis and nail disease associated with PsA and psoriasis, showing a significant difference versus placebo regardless of the dose [49].

Golimumab is a novel monoclonal antibody against TNFα, engineered in a transgenic mice model. The GO-REVEAL RCT demonstrated significant efficacy for treating psoriasis and PsA at week 14 as measured by ACR20 and PASI75 responses, and positive impact on quality of life, as reflected by significant improvements in the HAQ [54]. Efficacy was maintained at 5 years [55]; however, 31 % of patients had discontinued treatment after 5 years. Golimumab also proved to be effective in treating enthesitis at week 24 [54, 56]; however, dactylitis score was only significantly decreased with the 100-mg dose of golimumab compared to placebo [56]. Golimumab was also effective in treating nail disease [54].

### T cell modulators

#### Inhibition of T cell co-stimulatory MOLECULES

Abatacept is a fusion protein that binds to CD80 and CD86 and interferes with T cell signalling and activation, producing a reduction in inflammation. It has shown to be effective in the treatment of PsA and psoriasis (as assessed by ACR20, SF-36, HAQ and PASI scores); in addition, there were improvements in joint erosions, osteitis and synovitis [57]. It was noticed that skin response was inconsistent, and TNF-naïve patients showed greater responses.

Despite this, abatacept has failed to show efficacy in AS in a 24-week open-label study [58]. There have been no data to support its use in psoriatic arthritis with axial involvement.

Alefacept is a dimeric fusion protein that consists of the extracellular CD2-binding portion of the human leucocyte function antigen-3 (LFA-3) linked to the Fc portion of human IgG1 which acts as a T cell modulator. Studies have shown efficacy at week 12 for PASI75 [59–61] and DLQI [62]. When used in combination with methotrexate, the treatment was superior to MTX alone (as assessed by ACR20 and PASI50 scores at week 24) [63]. There are no data to support its efficacy in axial disease, dactylitis, enthesitis or nail disease.

Efalizumab is a recombinant humanised monoclonal antibody which binds to the CD11a subunit of LFA-1 and acts as an immunosuppressant by inhibiting lymphocyte activation and cell migration out of blood vessels into tissues, which was not superior to placebo in treating PsA [64], despite being proven effective in treating psoriasis [64, 65]. This drug was withdrawn in 2009 in Europe and the USA because of the increased risk of progressive multifocal leukoencephalopathy [64].

Itolizumab is the first humanised IgG1 monoclonal antibody, which targets selectively CD6, a marker involved in co-stimulation, adhesion and maturation of T cells, which was tested in psoriasis. The treatment was proven effective in improving the PASI75 score in several RCTs of patients with psoriasis [66, 67] and was licensed for use in India in 2013.

#### Phosphodiesterase 4 inhibitors

Apremilast is a phosphodiesterase 4 inhibitor, which increases levels of cAMP, resulting in decreased levels of proinflammatory cytokines.

This treatment was shown effective in PsA treatment [68]. The PALACE studies, a group of large phase III trials, have demonstrated its efficacy for ACR20 response at week 16 [69]. Continuous efficacy was noted for HAQ-DI, enthesitis and dactylitis (even if the improvement of the last two clinical features did not reach statistical significance at week 24). An improvement in skin psoriasis was also noted, although this was less significant. Efficacy on axial disease was not investigated.

A separate study looking at psoriasis found the treatment effective (significant improvements in PASI75, pruritus, DLQI and physician global assessment). There was also evidence for its role in nail disease treatment [70]. Apremilast is currently recommended by the National Psoriasis Foundation for skin and nail disease and skin, nail and joint disease, but with less enthusiasm and a lower ranking than adalimumab and etanercept [21].

### Anti-interleukin biologic agents

#### IL12/IL23 inhibition

Ustekinumab is a human monoclonal antibody directed against the p40 subunit of IL12/IL23, which has shown remarkable efficacy in the treatment of psoriasis [71–73], with associated sustained efficacy after 3 years of treatment; [74]. Efficacy has also been observed for the treatment of PsA, reflected in significant improvement in ACR20 at 12 and 24 weeks [75–77]. There was also significant improvement in quality of life, reflected by DLQI scores and HAQ-DI response at week 12 [78], together with ACR50, ACR70, Disease Activity Score (DAS28-CRP) responses and PASI75 at week 24 [76, 77]. Combined radiographic analysis of PSUMMIT 1 and 2 RCT showed significantly less radiographic progression in the active treatment groups [79].

Ustekinumab has proven efficacy in treating psoriatic nail disease [80] and for the treatment of enthesitis [76, 77]. However, whilst the PSUMMIT-1 RCT found that ustekinumab was efficacious in treating dactylitis and spondylitis reflected in the improvement in BASDAI score [76], PSUMMIT-2 did not find any significant difference between ustekinumab and placebo [75].

The ACCEPT RCT which compared head-to-head ustekinumab with etanercept found greater, but nonsignificant PASI75 response in the ustekinumab arm (67.5 % for the dose of 45 mg and 73.8 % for the dose of 90 mg vs. etanercept 56.8 %) [81].

#### IL17 inhibition

Secukinumab is an IL17A monoclonal antibody, with recognised efficacy in treating psoriasis. The ERASURE and JUNCTURE RCTs showed significant increase in PASI75 response and Modified-IGA response of 0 or 1 (indicating clear or almost clear psoriatic disease) versus placebo at week 12 [82, 83]. The treatment had also potential to improve the response when switched to intravenous administration [84]. There may be a role for secukinumab in the treatment of PsA, as proven by the FUTURE-1 [85] FUTURE-2 [86], as well as in treating skin disease and extra-articular manifestations (such as dactylitis and enthesitis) [85, 86]. Efficacy was observed regardless of use of prior TNF inhibitor. FUTURE-1 RCT also demonstrated significantly greater inhibition of joint structural damage at week 24, with responses maintained at week 52 [87].

Secukinumab may have a greater efficacy than the currently licensed biologics for psoriasis as proven by the FIXTURE RCT, which compared it to etanercept (*p* < 0.001) [82].

Brodalumab is a monoclonal antibody that targets and blocks the signalling pathway of interleukin receptors (IL17A, IL17F and IL23), which has been proven effective for psoriasis treatment. A phase 2 dose-ranging RCT has demonstrated significant increase in PASI75 response at week 12 regardless of dosage; in addition, significantly greater PASI90 response was seen in the 140- and 210-mg dose versus placebo [88]. The long-term efficacy of brodalumab for psoriasis is demonstrated by the sustained skin response from week 12 to week 120 [89]. There could be a potential role for brodalumab in the treatment of PsA as well: a phase 2 RCT showed a significant increase in ACR20 response at week 12, and the ACR20 response was similar in patients who had or had not received any previous biologic treatment [90]. This study did show significant improvement in the BASDAI score, but was not significant for the treatment of enthesitis and dactylitis.

Ixekizumab is a humanised monoclonal antibody against IL 17A, which has shown benefits for the treatment of psoriasis: in a phase 2 RCT, a significantly increased PASI75, PASI90 and PASI100 response was found at week 12, for the 75- and 150-mg dosage [91]. Another phase 2 RCT found potential efficacy for scalp psoriasis and nail disease improvement at week 12 [92].

#### IL6 inhibition

Tocilizumab is a monoclonal antibody against IL6, which has shown no efficacy in controlling symptoms of AS in two short-term RCTs, despite being effective in decreasing the CRP levels in the treatment arm [93]. No further studies in seronegative spondyloarthropathy were planned.

#### B cell depletion therapy

Rituximab is a chimeric monoclonal antibody against the protein CD20, which is primarily found on the surface of B cells. An exploratory open-label study found modest clinical improvement in ACR20 and enthesitis scores, but no improvement of PASI scores, dactylitis or BASDAI scores [94]. No other trials of Rituximab in PsA were planned.

## Small molecule inhibitors

### Janus kinase inhibitors

Tofacitinib is an oral inhibitor of Janus kinase, which has demonstrated significant benefit for the treatment of psoriasis: a phase 2b trial demonstrated a significant PASI75 response at week 12, regardless of dosage [95, 96]. There is no published date for tofacitinib in the treatment of PsA, but clinical trials are currently ongoing.

## Discussions

There is clear evidence from the literature data that the biologic therapeutic armamentarium for psoriasis and PsA is rapidly expanding. The majority of biologic treatments were first assessed for efficacy in psoriasis. Important observations emerged from recent clinical trials proving that the new biologic treatments for psoriasis have certain advantages when compared to the licensed ones.

Secukinumab and ustekinumab had greater efficacy compared to etanercept, as per two head-to-head studies in psoriasis. Alefacept showed sustained treatment benefit for a drug-free follow-up period of 12 weeks in patients with psoriasis (suggesting the possibility of intermittent treatment regimens), and itolizumab was associated with very prolonged drug-free remission (up to 5 years) [97].

In terms of treatment opportunities for patients with PsA, the new biologics reassuringly showed similar control of peripheral joint symptoms (indirect comparison showed the following percentages of ACR20 response: ustekinumab 90 mg, 42 %; secukinumab 300 mg, 54 %; brodalumab 280 mg, 64 %; abatacept 10 mg/kg, 48 %; apremilast 20 mg daily, 43.5 %, which is comparable to infliximab 5 mg/kg, 65 %; certolizumab 200 mg e.o.w., 58 %; golimumab 100 mg monthly, 61 %; adalimumab 40 mg e.o.w, 58 %; and etanercept 25 mg twice weekly, 59 %). Different aspects of the disease activity, such as dactylitis and enthesitis, were effectively controlled by anti-TNF therapy, and also by ustekinumab and secukinumab. The axial involvement also responded to therapy with ustekinumab and secukinumab, and the nail involvement, enthesitis and dactylitis associated with PsA were all improved with treatment with apremilast and sekukinumab (along with infliximab, certolizumab, etanercept, adalimumab and golimumab).

Optimising therapy for those patients who failed anti-TNF treatments is one of the main challenges of managing patients with severe, longstanding PsA. In order to determine the efficacy of new biologics in this category of patients, different strategies of optimising the doses were assessed in clinical trials with secukinumab. The dose adjustment (intravenous loading dose and use of the 300-mg dose monthly) showed the best response in PsA patients previously treated with anti-TNF therapy.

Recent data from the NOR-DMARD cohort showed that the response to the second anti-TNF, in patients with PsA who failed the first anti-TNF, is significantly lower [14]; the use of other biologic treatments with different mechanisms of action is therefore currently considered a better option. In comparison with RA, and in both AS and PsA, the retention rates of first anti-TNF treatment and the response to the second anti-TNF are higher, although these are decreased compared to the first anti-TNF agent [98]. Therefore, the switch to the second anti-TNF might therefore be recommended in most cases when no other (biologic) treatments are available.

Table 1 includes a summary of biologic treatments and their efficacy for different clinical manifestations in PsA and psoriasis, using the following level of evidence classification (Oxford Centre of Evidence-based Medicine, 2009):

**Table 1:** Biologic treatment effectiveness in relation to various disease manifestations

Treatment	Peripheral arthritis	Sacroiliitis and spinal disease	Enthesitis	Dactylitis	Nail involvement	Skin psoriasis
*TNF inhibitors*						
ADALIMUMAB	YES (*1a)				YES (*1a)	YES (*1a)
ETANERCEPT	YES (*1a)	YES (*1a)	YES (*1a)	YES (*1a)	YES (*1a)	YES (*1a)
CERTOLIZUMAB	YES (*1a)	YES (*1a)	YES (*1a)	YES (*1a)	YES (*1a)	YES (*1a)
GOLIMUMAB	YES (*1a)	YES (*1a)	YES (*1a)	YES (*1a)	YES (*1a)	YES (*1a)
INFLIXIMAB	YES (*1a)	YES (*1a)	YES (*1a)	YES (*1a)	YES (*1a)	YES (*1a)
*T cell modulators*						
ABATACEPT	YES (*1b)	NO (*1b)-study in AS				YES (*1b)
ITOLIZUMAB	Planned studies	Planned studies	Planned studies	Planned studies	Planned studies	YES (*1b)
ALEFACEPT						YES (*1a)
EFALIZUMAB (withdrawn)	NO (*1b)					YES (*1a)
*Phosphodiesterase 4* *Inhibitors*						
APREMILAST	YES (*1a)		YES (*1b)	YES (*1b)	YES (*1b)	YES (*1a)
*IL inhibition*						
USTEKINUMAB	YES (*1a)	YES (*1b)	YES (*1b)	YES (*1b)		YES (*1a)
BRODALUMAB	YES (*1b)	YES (*1b)	NO (*1b)	NO (*1b)		
IXEKIZUMAB	Ongoing study		Ongoing study	Ongoing study	Ongoing study	YES (*1a)
SECUKINUMAB	YES (*1a)	YES (*1a)	YES (*1a)	YES (*1a)		
TOCILIZUMAB	YES (*4)	NO (*1b)				YES (pustular psoriasis) (*4)
*B cell depletion therapy*						
RITUXIMAB	NO (*1b)		YES (*1b)	NO (*1b)		
*Small molecule inhibitors*						
TOFACITINIB	Ongoing studies	Under recruitment in AS	Ongoing studies	Ongoing studies		YES (*1a)

* Level of evidence

1a: Systematic reviews (with homogeneity) of randomised controlled trials
1b: Individual randomised controlled trials (with narrow confidence interval)
1c: “All or none” randomised controlled trials
2a: Systematic reviews (with homogeneity) of cohort studies
2b: Individual cohort study or low-quality randomised controlled trials (e.g. <80 % follow-up)
2c: “Outcomes” research; ecological studies
3a: Systematic review (with homogeneity) of case–control studies
3b: Individual case–control study
4: Case series (and poor quality cohort and case–control studies)
5: Expert opinion without explicit critical appraisal, or based on physiology, bench research or “first principles”

It is difficult to establish an algorithm for sequential biologic treatment in PsA patients who failed the first biologic (usually an anti-TNF drug), due to of lack of evidence of efficacy of the majority of new drugs as second-line biologic therapies.

Based on the information available, we can make the following recommendations for treatment of PsA patients who failed one anti-TNF treatment: ustekinumab can be used as second-line biologic in psoriatic and PsA patients who failed TNF treatments (level of evidence 1b); sekukinumab at higher dose (300 mg monthly) and with additional IV loading dose is effective in PsA patients who failed anti-TNF therapy (level of evidence 1b); and the use of a second anti-TNF therapy can be effective in patients who failed the first anti-TNF treatment (certolizumab and golimumab, level of evidence 1b; infliximab and adalimumab and etanercept, level of evidence 2b).

In summary, this review highlighted that the number of biologic treatments for PsA and psoriasis increased significantly in the recent years and will probably lead in the future to the licensing of new therapies. Given the heterogeneity of both PsA and psoriasis, the treatments should be tailored to individual cases. Further research into both prognostic biomarkers and patient stratification is required to allow clinicians the possibility to make better use of the various biologic treatment options available.

## References

[1] Gladman DD, Mease PJ (2006). Towards international guidelines for the management of psoriatic arthritis. J Rheumatol.

[2] Gossec L, Smolen JS, Gaujoux-Viala C, Ash Z, Marzo-Ortega H, van der Heijde D (2012). European league against rheumatism recommendations for the management of psoriatic arthritis with pharmacological therapies. Ann Rheum Dis.

[3] Ash Z, Gaujoux-Viala C, Gossec L, Hensor EM, FitzGerald O, Winthrop K (2012). A systematic literature review of drug therapies for the treatment of psoriatic arthritis: current evidence and meta-analysis informing the EULAR recommendations for the management of psoriatic arthritis. Ann Rheum Dis.

[4] Menter A, Tyring SK, Gordon K, Kimball AB, Leonardi CL, Langley RG (2008). Adalimumab therapy for moderate to severe psoriasis: a randomized, controlled phase III trial. J Am Acad Dermatol.

[5] Mease PJ, Gladman DD, Ritchlin CT, Ruderman EM, Steinfeld SD, Choy EH (2005). Adalimumab for the treatment of patients with moderately to severely active psoriatic arthritis: results of a double-blind, randomized, placebo-controlled trial. Arthritis Rheum.

[6] Genovese MC, Mease PJ, Thomson GT, Kivitz AJ, Perdok RJ, Weinberg MA (2007). Safety and efficacy of adalimumab in treatment of patients with psoriatic arthritis who had failed disease modifying antirheumatic drug therapy. J Rheumatol.

[7] Revicki DA, Willian MK, Menter A, Gordon KB, Kimball AB, Leonardi CL (2007). Impact of adalimumab treatment on patient-reported outcomes: results from a phase III clinical trial in patients with moderate to severe plaque psoriasis. J Dermatol Treat.

[8] Gladman DD, Mease PJ, Cifaldi MA, Perdok RJ, Sasso E, Medich J (2007). Adalimumab improves joint-related and skin-related functional impairment in patients with psoriatic arthritis: patient-reported outcomes of the adalimumab effectiveness in psoriatic arthritis trial. Ann Rheum Dis.

[9] Saurat JH, Stingl G, Dubertret L, Papp K, Langley RG, Ortonne JP (2008). Efficacy and safety results from the randomized controlled comparative study of adalimumab versus methotrexate versus placebo in patients with psoriasis (CHAMPION). Br J Dermatol.

[10] Karanikolas GN, Koukli EM, Katsalira A, Arida A, Petrou D, Komninou E (2011). Adalimumab or cyclosporine as monotherapy and in combination in severe psoriatic arthritis: results from a prospective 12-month nonrandomized unblinded clinical trial. J Rheumatol.

[11] Atteno M, Peluso R, Costa L, Padula S, Iervolino S, Caso F (2010). Comparison of effectiveness and safety of infliximab, etanercept, and adalimumab in psoriatic arthritis patients who experienced an inadequate response to previous disease-modifying antirheumatic drugs. Clin Rheumatol.

[12] Fenix-Caballero S, Alegre-del Rey EJ, Castano-Lara R, Puigventos-Latorre F, Borrero-Rubio JM, Lopez-Vallejo JF (2013). Direct and indirect comparison of the efficacy and safety of adalimumab, etanercept, infliximab and golimumab in psoriatic arthritis. J Clin Pharm Ther.

[13] Thorlund K, Druyts E, Avina-Zubieta JA, Mills EJ (2012). Anti-tumor necrosis factor (TNF) drugs for the treatment of psoriatic arthritis: an indirect comparison meta-analysis. Biol Targets Ther.

[14] Fagerli KM, Lie E, van der Heijde D, Heiberg MS, Kalstad S, Rodevand E (2013). Switching between TNF inhibitors in psoriatic arthritis: data from the NOR-DMARD study. Ann Rheum Dis.

[15] Glintborg B, Ostergaard M, Krogh NS, Andersen MD, Tarp U, Loft AG (2013). Clinical response, drug survival, and predictors thereof among 548 patients with psoriatic arthritis who switched tumor necrosis factor alpha inhibitor therapy: results from the Danish Nationwide DANBIO registry. Arthritis Rheum.

[16] Gladman DD, Investigators AS, Sampalis JS, Illouz O, Guerette B (2010). Responses to adalimumab in patients with active psoriatic arthritis who have not adequately responded to prior therapy: effectiveness and safety results from an open-label study. J Rheumatol.

[17] Leonardi C, Langley RG, Papp K, Tyring SK, Wasel N, Vender R (2011). Adalimumab for treatment of moderate to severe chronic plaque psoriasis of the hands and feet: efficacy and safety results from REACH, a randomized, placebo-controlled, double-blind trial. Arch Dermatol.

[18] Van den Bosch F, Manger B, Goupille P, McHugh N, Rodevand E, Holck P (2010). Effectiveness of adalimumab in treating patients with active psoriatic arthritis and predictors of good clinical responses for arthritis, skin and nail lesions. Ann Rheum Dis.

[19] Manger B, Lorenz H, Sprekeler R (2008). Adalimumab (Humira) improves the signs and symptoms of joint, skin and nail manifestations of psoriatic arthritis (PsA): results from a German database of PsA patients receiving adalimumab. Ann Rheum Dis.

[20] Rigopoulos D, Gregoriou S, Lazaridou E, Belyayeva E, Apalla Z, Makris M (2010). Treatment of nail psoriasis with adalimumab: an open label unblinded study. J Eur Acad Dermatol Venereol JEADV.

[21] Crowley JJ, Weinberg JM, Wu JJ, Robertson AD, Van Voorhees AS (2015). Treatment of nail psoriasis: best practice recommendations from the medical board of the national psoriasis foundation. JAMA Dermatol.

[22] Braun J, Rudwaleit M, Kary S, Kron M, Wong RL, Kupper H (2010). Clinical manifestations and responsiveness to adalimumab are similar in patients with ankylosing spondylitis with and without concomitant psoriasis. Rheumatology.

[23] Cohen Barak E, Kerner M, Rozenman D, Ziv M (2015). Combination therapy of cyclosporine and anti-tumor necrosis factor alpha in psoriasis: a case series of ten patients. Dermatol Ther.

[24] Mease PJ, Goffe BS, Metz J, VanderStoep A, Finck B, Burge DJ (2000). Etanercept in the treatment of psoriatic arthritis and psoriasis: a randomised trial. Lancet.

[25] Gottlieb AB, Matheson RT, Lowe N, Krueger GG, Kang S, Goffe BS, et al. (2003) A randomized trial of etanercept as monotherapy for psoriasis. Arch Dermatol 139(12):1627–1632; discussion 32 10.1001/archderm.139.12.1627 14676082

[26] Leonardi CL, Powers JL, Matheson RT, Goffe BS, Zitnik R, Wang A (2003). Etanercept as monotherapy in patients with psoriasis. N Engl J Med.

[27] Krueger GG, Langley RG, Finlay AY, Griffiths CE, Woolley JM, Lalla D (2005). Patient-reported outcomes of psoriasis improvement with etanercept therapy: results of a randomized phase III trial. Br J Dermatol.

[28] Papp KA, Tyring S, Lahfa M, Prinz J, Griffiths CE, Nakanishi AM (2005). A global phase III randomized controlled trial of etanercept in psoriasis: safety, efficacy, and effect of dose reduction. Br J Dermatol.

[29] Tyring S, Gottlieb A, Papp K, Gordon K, Leonardi C, Wang A (2006). Etanercept and clinical outcomes, fatigue, and depression in psoriasis: double-blind placebo-controlled randomised phase III trial. Lancet.

[30] van de Kerkhof PC, Segaert S, Lahfa M, Luger TA, Karolyi Z, Kaszuba A (2008). Once weekly administration of etanercept 50 mg is efficacious and well tolerated in patients with moderate-to-severe plaque psoriasis: a randomized controlled trial with open-label extension. Br J Dermatol.

[31] Reich K, Segaert S, Van de Kerkhof P, Durian C, Boussuge MP, Paolozzi L (2009). Once-weekly administration of etanercept 50 mg improves patient-reported outcomes in patients with moderate-to-severe plaque psoriasis. Dermatology.

[32] Mease PJ, Kivitz AJ, Burch FX, Siegel EL, Cohen SB, Ory P (2004). Etanercept treatment of psoriatic arthritis: safety, efficacy, and effect on disease progression. Arthritis Rheum.

[33] Mease PJ, Kivitz AJ, Burch FX, Siegel EL, Cohen SB, Ory P (2006). Continued inhibition of radiographic progression in patients with psoriatic arthritis following 2 years of treatment with etanercept. J Rheumatol.

[34] Lubrano E, Spadaro A, Marchesoni A, Olivieri I, Scarpa R, D’Angelo S (2011). The effectiveness of a biologic agent on axial manifestations of psoriatic arthritis. A 12 months observational study in a group of patients treated with etanercept. Clin Exp Rheumatol.

[35] Sterry W, Ortonne JP, Kirkham B, Brocq O, Robertson D, Pedersen RD (2010). Comparison of two etanercept regimens for treatment of psoriasis and psoriatic arthritis: PRESTA randomised double blind multicentre trial. BMJ.

[36] Ortonne JP, Paul C, Berardesca E, Marino V, Gallo G, Brault Y (2013). A 24-week randomized clinical trial investigating the efficacy and safety of two doses of etanercept in nail psoriasis. Br J Dermatol.

[37] Luger TA, Barker J, Lambert J, Yang S, Robertson D, Foehl J (2009). Sustained improvement in joint pain and nail symptoms with etanercept therapy in patients with moderate-to-severe psoriasis. J Eur Acad Dermatol Venereol JEADV.

[38] Torii H, Nakagawa H (2010). Infliximab monotherapy in Japanese patients with moderate-to-severe plaque psoriasis and psoriatic arthritis. A randomized, double-blind, placebo-controlled multicenter trial. J Dermatol Sci.

[39] Antoni CE, Kavanaugh A, Kirkham B, Tutuncu Z, Burmester GR, Schneider U (2005). Sustained benefits of infliximab therapy for dermatologic and articular manifestations of psoriatic arthritis: results from the infliximab multinational psoriatic arthritis controlled trial (IMPACT). Arthritis Rheum.

[40] Reich K, Nestle FO, Papp K, Ortonne JP, Evans R, Guzzo C (2005). Infliximab induction and maintenance therapy for moderate-to-severe psoriasis: a phase III, multicentre, double-blind trial. Lancet.

[41] Menter A, Feldman SR, Weinstein GD, Papp K, Evans R, Guzzo C (2007). A randomized comparison of continuous versus intermittent infliximab maintenance regimens over 1 year in the treatment of moderate-to-severe plaque psoriasis. J Am Acad Dermatol.

[42] Kavanaugh A, Antoni C, Krueger GG, Yan S, Bala M, Dooley LT (2006). Infliximab improves health related quality of life and physical function in patients with psoriatic arthritis. Ann Rheum Dis.

[43] Kavanaugh A, Krueger GG, Beutler A, Guzzo C, Zhou B, Dooley LT (2007). Infliximab maintains a high degree of clinical response in patients with active psoriatic arthritis through 1 year of treatment: results from the IMPACT 2 trial. Ann Rheum Dis.

[44] van der Heijde D, Kavanaugh A, Gladman DD, Antoni C, Krueger GG, Guzzo C (2007). Infliximab inhibits progression of radiographic damage in patients with active psoriatic arthritis through 1 year of treatment: results from the induction and maintenance psoriatic arthritis clinical trial 2. Arthritis Rheum.

[45] Barker J, Hoffmann M, Wozel G, Ortonne JP, Zheng H, van Hoogstraten H (2011). Efficacy and safety of infliximab versus methotrexate in patients with moderate-to-severe plaque psoriasis: results of an open-label, active-controlled, randomized trial (RESTORE1). Br J Dermatol.

[46] Baranauskaite A, Raffayova H, Kungurov NV, Kubanova A, Venalis A, Helmle L (2012). Infliximab plus methotrexate is superior to methotrexate alone in the treatment of psoriatic arthritis in methotrexate-naive patients: the RESPOND study. Ann Rheum Dis.

[47] Gottlieb AB, Kalb RE, Blauvelt A, Heffernan MP, Sofen HL, Ferris LK (2012). The efficacy and safety of infliximab in patients with plaque psoriasis who had an inadequate response to etanercept: results of a prospective, multicenter, open-label study. J Am Acad Dermatol.

[48] Reich K, Ortonne JP, Gottlieb AB, Terpstra IJ, Coteur G, Tasset C (2012). Successful treatment of moderate to severe plaque psoriasis with the PEGylated Fab’ certolizumab pegol: results of a phase II randomized, placebo-controlled trial with a re-treatment extension. Br J Dermatol.

[49] Mease PJ, Fleischmann R, Deodhar AA, Wollenhaupt J, Khraishi M, Kielar D (2014). Effect of certolizumab pegol on signs and symptoms in patients with psoriatic arthritis: 24-week results of a phase 3 double-blind randomised placebo-controlled study (RAPID-PsA). Ann Rheum Dis.

[50] Mease PJ, Fleischmann R, Wollenhaupt J, Deodhar A, Gladman D, Stach C (2014). 210. Effect of certolizumab pegol over 48 weeks on signs and symptoms in patients with psoriatic arthritis with and without prior tumor necrosis factor inhibitor exposure. Rheumatology.

[51] Gladman D, Fleischmann R, Coteur G, Woltering F, Mease PJ (2014). Effect of certolizumab pegol on multiple facets of psoriatic arthritis as reported by patients: 24-week patient-reported outcome results of a phase III, multicenter study. Arthritis Care Res.

[52] Kavanaugh A, Gladman D, van der Heijde D, Purcaru O, Mease P (2015). Improvements in productivity at paid work and within the household, and increased participation in daily activities after 24 weeks of certolizumab pegol treatment of patients with psoriatic arthritis: results of a phase 3 double-blind randomised placebo-controlled study. Ann Rheum Dis.

[53] van der Heijde D, Fleischmann R, Wollenhaupt J, Deodhar A, Kielar D, Woltering F (2014). Effect of different imputation approaches on the evaluation of radiographic progression in patients with psoriatic arthritis: results of the RAPID-PsA 24-week phase III double-blind randomised placebo-controlled study of certolizumab pegol. Ann Rheum Dis.

[54] Kavanaugh A, McInnes I, Mease P, Krueger GG, Gladman D, Gomez-Reino J (2009). Golimumab, a new human tumor necrosis factor alpha antibody, administered every 4 weeks as a subcutaneous injection in psoriatic arthritis: 24-week efficacy and safety results of a randomized, placebo-controlled study. Arthritis Rheum.

[55] Kavanaugh A, McInnes IB, Mease P, Krueger GG, Gladman D, van der Heijde D (2014). Clinical efficacy, radiographic and safety findings through 5 years of subcutaneous golimumab treatment in patients with active psoriatic arthritis: results from a long-term extension of a randomised, placebo-controlled trial (the GO-REVEAL study). Ann Rheum Dis.

[56] Kavanaugh A, Mease P (2012). Treatment of psoriatic arthritis with tumor necrosis factor inhibitors: longer-term outcomes including enthesitis and dactylitis with golimumab treatment in the longterm extension of a randomized, placebo-controlled study (GO-REVEAL). J Rheumatol Suppl.

[57] Mease P, Genovese MC, Gladstein G, Kivitz AJ, Ritchlin C, Tak PP (2011). Abatacept in the treatment of patients with psoriatic arthritis: results of a 6-month, multicenter, randomized, double-blind, placebo-controlled, phase II trial. Arthritis Rheum.

[58] Song IH, Heldmann F, Rudwaleit M, Haibel H, Weiss A, Braun J (2011). Treatment of active ankylosing spondylitis with abatacept: an open-label, 24-week pilot study. Ann Rheum Dis.

[59] Krueger GG, Papp KA, Stough DB, Loven KH, Gulliver WP, Ellis CN (2002). A randomized, double-blind, placebo-controlled phase III study evaluating efficacy and tolerability of 2 courses of alefacept in patients with chronic plaque psoriasis. J Am Acad Dermatol.

[60] Lebwohl M, Christophers E, Langley R, Ortonne JP, Roberts J, Griffiths CE (2003). An international, randomized, double-blind, placebo-controlled phase 3 trial of intramuscular alefacept in patients with chronic plaque psoriasis. Arch Dermatol.

[61] Ortonne JP (2003). Clinical response to alefacept: results of a phase 3 study of intramuscular administration of alefacept in patients with chronic plaque psoriasis. J Eur Acad Dermatol Venereol JEADV.

[62] Feldman SR, Menter A, Koo JY (2004). Improved health-related quality of life following a randomized controlled trial of alefacept treatment in patients with chronic plaque psoriasis. Br J Dermatol.

[63] Mease PJ, Gladman DD, Keystone EC, Alefacept in Psoriatic Arthritis Study G (2006). Alefacept in combination with methotrexate for the treatment of psoriatic arthritis: results of a randomized, double-blind, placebo-controlled study. Arthritis Rheum.

[64] Papp KA, Caro I, Leung HM, Garovoy M, Mease PJ (2007). Efalizumab for the treatment of psoriatic arthritis. J Cutan Med Surg.

[65] Lotti T, Chimenti S, Katsambas A, Ortonne JP, Dubertret L, Licu D (2010). Efficacy and safety of efalizumab in patients with moderate-to-severe plaque psoriasis resistant to previous anti-psoriatic treatment: results of a multicentre, open-label, phase IIIb/IV trial. Arch Drug Inf.

[66] Krupashankar DS, Dogra S, Kura M, Saraswat A, Budamakuntla L, Sumathy TK (2014). Efficacy and safety of itolizumab, a novel anti-CD6 monoclonal antibody, in patients with moderate to severe chronic plaque psoriasis: results of a double-blind, randomized, placebo-controlled, phase-III study. J Am Acad Dermatol.

[67] Dogra S, Budamakuntla L, Srinivas CR, Khopkar U, Gupta S (2015). Long-term efficacy and safety of itolizumab in patients with moderate-to-severe chronic plaque psoriasis: a double-blind, randomized-withdrawal, placebo-controlled study. J Am Acad Dermatol.

[68] Schett G, Wollenhaupt J, Papp K, Joos R, Rodrigues JF, Vessey AR (2012). Oral apremilast in the treatment of active psoriatic arthritis: results of a multicenter, randomized, double-blind, placebo-controlled study. Arthritis Rheum.

[69] Kavanaugh A, Mease PJ, Gomez-Reino JJ, Adebajo AO, Wollenhaupt J, Gladman DD (2014). Treatment of psoriatic arthritis in a phase 3 randomised, placebo-controlled trial with apremilast, an oral phosphodiesterase 4 inhibitor. Ann Rheum Dis.

[70] Papp K, Cather JC, Rosoph L, Sofen H, Langley RG, Matheson RT (2012). Efficacy of apremilast in the treatment of moderate to severe psoriasis: a randomised controlled trial. Lancet.

[71] Leonardi CL, Kimball AB, Papp KA, Yeilding N, Guzzo C, Wang Y (2008). Efficacy and safety of ustekinumab, a human interleukin-12/23 monoclonal antibody, in patients with psoriasis: 76-week results from a randomised, double-blind, placebo-controlled trial (PHOENIX 1). Lancet.

[72] Papp KA, Langley RG, Lebwohl M, Krueger GG, Szapary P, Yeilding N (2008). Efficacy and safety of ustekinumab, a human interleukin-12/23 monoclonal antibody, in patients with psoriasis: 52-week results from a randomised, double-blind, placebo-controlled trial (PHOENIX 2). Lancet.

[73] Tsai TF, Ho JC, Song M, Szapary P, Guzzo C, Shen YK (2011). Efficacy and safety of ustekinumab for the treatment of moderate-to-severe psoriasis: a phase III, randomized, placebo-controlled trial in Taiwanese and Korean patients (PEARL). J Dermatol Sci.

[74] Kimball AB, Gordon KB, Fakharzadeh S, Yeilding N, Szapary PO, Schenkel B (2012). Long-term efficacy of ustekinumab in patients with moderate-to-severe psoriasis: results from the PHOENIX 1 trial through up to 3 years. Br J Dermatol.

[75] Ritchlin C, Rahman P, Kavanaugh A, McInnes IB, Puig L, Li S (2014). Efficacy and safety of the anti-IL-12/23 p40 monoclonal antibody, ustekinumab, in patients with active psoriatic arthritis despite conventional non-biological and biological anti-tumour necrosis factor therapy: 6-month and 1-year results of the phase 3, multicentre, double-blind, placebo-controlled, randomised PSUMMIT 2 trial. Ann Rheum Dis.

[76] McInnes IB, Kavanaugh A, Gottlieb AB, Puig L, Rahman P, Ritchlin C (2013). Efficacy and safety of ustekinumab in patients with active psoriatic arthritis: 1 year results of the phase 3, multicentre, double-blind, placebo-controlled PSUMMIT 1 trial. Lancet.

[77] Gottlieb A, Menter A, Mendelsohn A, Shen YK, Li S, Guzzo C (2009). Ustekinumab, a human interleukin 12/23 monoclonal antibody, for psoriatic arthritis: randomised, double-blind, placebo-controlled, crossover trial. Lancet.

[78] Kavanaugh A, Menter A, Mendelsohn A, Shen YK, Lee S, Gottlieb AB (2010). Effect of ustekinumab on physical function and health-related quality of life in patients with psoriatic arthritis: a randomized, placebo-controlled, phase II trial. Curr Med Res Opin.

[79] Kavanaugh A, Ritchlin C, Rahman P, Puig L, Gottlieb AB, Li S (2014). Ustekinumab, an anti-IL-12/23 p40 monoclonal antibody, inhibits radiographic progression in patients with active psoriatic arthritis: results of an integrated analysis of radiographic data from the phase 3, multicentre, randomised, double-blind, placebo-controlled PSUMMIT-1 and PSUMMIT-2 trials. Ann Rheum Dis.

[80] Rich P, Bourcier M, Sofen H, Fakharzadeh S, Wasfi Y, Wang Y (2014). Ustekinumab improves nail disease in patients with moderate-to-severe psoriasis: results from PHOENIX 1. Br J Dermatol.

[81] Young MS, Horn EJ, Cather JC (2011). The ACCEPT study: ustekinumab versus etanercept in moderate-to-severe psoriasis patients. Expert Rev Clin Immunol.

[82] Langley RG, Elewski BE, Lebwohl M, Reich K, Griffiths CEM, Papp K (2014). Secukinumab in plaque psoriasis—results of two phase 3 trials. N Engl J Med.

[83] Paul C, Lacour JP, Tedremets L, Kreutzer K, Jazayeri S, Adams S (2015). Efficacy, safety and usability of secukinumab administration by autoinjector/pen in psoriasis: a randomized, controlled trial (JUNCTURE). J Eur Acad Dermatol Venereol.

[84] Thaci D, Humeniuk J, Frambach Y, Bissonnette R, Goodman JJ, Shevade S et al (2015) Secukinumab in psoriasis: randomized, controlled phase 3 trial results assessing the potential to improve treatment response in partial responders (STATURE). Br J Dermatol 10.1111/bjd.13814 25823958

[85] Mease P, McInnes I, Kirkham B, Kavanaugh A, Rahman P, van der Heijde D et al (2014) Secukinumab, a human anti–interleukin-17A monoclonal antibody, improves active psoriatic arthritis and inhibits radiographic progression: efficacy and safety data from a phase 3 randomized, multicenter, double-blind, Placebo-Controlled Study. ACR 2014; Boston

[86] McInnes I, Mease P, Kirkham B, Kavanaugh A, Ritchlin C, Rahman P et al (2014) Secukinumab, a human anti-interleukin-17A monoclonal antibody, improves active psoriatic arthritis: 24-week efficacy and safety data from a phase 3 randomized, multicenter, double-blind, Placebo-Controlled Study using subcutaneous dosing. ACR 2014; Boston

[87] van der Heijde D, Landewé R, Mease P, McInnes I, Conaghan P, Pricop L et al (2014) Secukinumab, a monoclonal antibody to interleukin-17A, provides significant and sustained inhibition of joint structural damage in active psoriatic arthritis regardless of prior TNF inhibitors or concomitant methotrexate: a phase 3 randomized, double-blind, Placebo-Controlled Study. ACR 2014; Boston

[88] Papp KA, Leonardi C, Menter A, Ortonne J-P, Krueger JG, Kricorian G (2012). Brodalumab, an anti–interleukin-17–receptor antibody for psoriasis. N Engl J Med.

[89] Papp K, Leonardi C, Menter A, Thompson EH, Milmont CE, Kricorian G (2014). Safety and efficacy of brodalumab for psoriasis after 120 weeks of treatment. J Am Acad Dermatol.

[90] Mease PJ, Genovese MC, Greenwald MW, Ritchlin CT, Beaulieu AD, Deodhar A (2014). Brodalumab, an anti-IL17RA monoclonal antibody, in psoriatic arthritis. N Engl J Med.

[91] Leonardi C, Matheson R, Zachariae C, Cameron G, Li L, Edson-Heredia E (2012). Anti–interleukin-17 monoclonal antibody ixekizumab in chronic plaque psoriasis. N Engl J Med.

[92] Langley RG, Rich P, Menter A, Krueger G, Goldblum O, Dutronc Y et al (2015) Improvement of scalp and nail lesions with ixekizumab in a phase 2 trial in patients with chronic plaque psoriasis. J Eur Acad Dermatol Venereol JEADV 10.1111/jdv.12996 25693783

[93] Sieper J, Porter-Brown B, Thompson L, Harari O, Dougados M (2014). Assessment of short-term symptomatic efficacy of tocilizumab in ankylosing spondylitis: results of randomised, placebo-controlled trials. Ann Rheum Dis.

[94] Jimenez-Boj E, Stamm TA, Sadlonova M, Rovensky J, Raffayova H, Leeb B (2012). Rituximab in psoriatic arthritis: an exploratory evaluation. Ann Rheum Dis.

[95] Papp KA, Menter A, Strober B, Langley RG, Buonanno M, Wolk R (2012). Efficacy and safety of tofacitinib, an oral Janus kinase inhibitor, in the treatment of psoriasis: a phase 2b randomized placebo-controlled dose-ranging study. Br J Dermatol.

[96] Menter A, Papp KA, Tan H, Tyring S, Wolk R, Buonanno M (2014). Efficacy of tofacitinib, an oral janus kinase inhibitor, on clinical signs of moderate-to-severe plaque psoriasis in different body regions. J Drugs Dermatol.

[97] Budamakuntla L, Madaiah M, Sarvajnamurthy S, Kapanigowda S (2015). Itolizumab provides sustained remission in plaque psoriasis: a 5-year follow-up experience. Clin Exp Dermatol.

[98] Biggioggero M, Favalli EG (2014). 10-year drug survival of anti-TNF agents in the treatment of inflammatory arthritides. Drug Dev Res.

